# Efficacy of a Zinc Lactate Mouthwash and Tongue Scraping in the Reduction of Intra-Oral Halitosis: A Single-blind, Controlled, Crossover Clinical Trial—A Pilot Study

**DOI:** 10.3390/jcm10235532

**Published:** 2021-11-26

**Authors:** Agata Dudzik, Sarkis Sozkes, Ewa Michalak, Iwona Olszewska-Czyz

**Affiliations:** 1 Periodontology, Prophylaxis and Oral Pathology Department, Medical Faculty, Jagiellonian University, 31155 Krakow, Poland; agata.skrzypek@uj.edu.pl (A.D.); ewa3.michalak@uj.edu.pl (E.M.); 2 Biomedical Engineering Biomaterials Department, Tekirdag Namik Kemal University, 59860 Tekirdag, Turkey; ssozkes@nku.edu.tr

**Keywords:** intra-oral halitosis, breath odor, volatile sulfur compounds

## Abstract

Intra-oral halitosis is defined as an unpleasant odor that comes out of the mouth. The aim of this study was to investigate the effect of zinc lactate mouthwash and tongue scraping on intra-oral halitosis. The study was conducted on 60 volunteers that were divided into two groups and fol-lowed two types of 14-day oral hygiene protocols on a cross-over basis after a 7-day wash-out period. One protocol was based on tooth brushing only, while the other was based on additional mouth rinsing with a zinc lactate product and tongue scraping. Morning mouth breath was as-sessed organoleptic and by volatile sulfur compound concentrations. The highest mean organo-leptic and volatile sulfur compound measurement values were found in the tooth brushing without mouth washing and tongue scraping oral hygiene protocol (*p* < 0.05). The zinc lactate mouthwash combined with tongue scraping appears to be an important hygienic procedure to reduce breath odor.

## 1. Introduction

### 1.1. Background

Intra-oral halitosis is defined as an unpleasant odor that comes out of the mouth. Several synonyms of intra-oral halitosis are described in the literature: bad breath, fetor ex ore, fetor oris and oral malodor [1]. It is estimated that this condition affects 25% to 50% of people worldwide [2]. Many different factors can cause intra-oral halitosis. Ninety percent originates from the head (mouth, sinuses, tonsils), and about 10% of the causes are systemic. Unpleasant odors are represented by fragrant volatile compounds present in the exhaled air, and among them, the most frequently mentioned are Volatile Sulfur Compounds (VSC) such as hydrogen sulfide (H_2_S), methyl mercaptan (CH_3_SH) and dimethyl sulfide ((CH_3_)_2_S) [3]. The most common methods used to diagnose intra-oral halitosis are organoleptic evaluation and gas chromatography [4]. The most likely origin of oral malodor is the accumulation of food debris with subsequent metabolism by bacteria on the dorsum part of the tongue. The anatomy of the tongue offers numerous ecological niches for various bacteria to settle on its surface [2,5].

The success of any intra-oral halitosis intervention appears to depend on the reduction in VSC levels in the exhaled air. Many strategies were developed for the reduction in halitosis. These protocols typically require physical or chemical methods [1]. Mechanical approaches to clean the dorsum of the tongue, such as tongue brushing or tongue scraping, have the potential to successfully reduce tongue coating thickness and oral malodor, yet the limitations of mechanical methods to effectively reach and remove VSC-producing bacteria from all oral ecological sites are acknowledged [6]. The possibility that mouth rinses may be more effective in reaching the less accessible parts of the oral cavity and ease of use has led to the development of mouth rinses specially designed for the treatment of intra-oral halitosis. Different oral care products for reducing oral malodor were developed, and they contain antibacterial substances such as chlorhexidine (CHX) and cetylpyridinium chloride (CPC). These products were shown to inhibit the mechanism of bacteria and reduce the bacterial load, yet CHX has the disadvantages of increased tooth staining [7,8]. Most of the studies show that mouthwashes containing zinc ions can induce short- and long-term neutralizing effects with fewer side effects [7,8]. Zinc added to mouthwashes can inhibit the formation of VSC and reduce intra-oral halitosis. Moreover, zinc ions interact with the sulfur in order to form insoluble sulfide. It can cause a reduction in the VSC in the exhaled air. In addition, zinc phosphate restraints microbial activity by reacting with H_2_S gas [2,7,8]. The study of Srisilapanan et al. tested the combination of toothpaste and mouth rinse containing 0.14% zinc lactate, and they reported that this combination reduced the three volatile gases and total VSC at all assessment times [8].

### 1.2. Objectives

The study design aimed to investigate the effect of the use of the tongue scraper and the zinc lactate-containing mouthwash on intra-oral halitosis.

### 1.3. Trial Design

**Figure 1:**
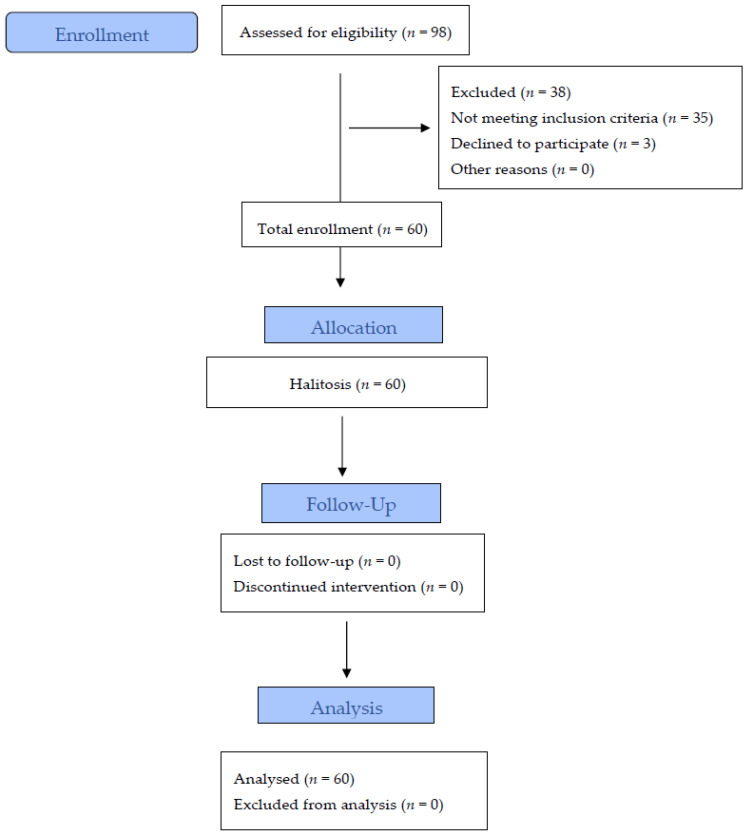
Trial Diagram.

The trial was a single-center, prospective, clinical, controlled, cross-over, single-blind trial conducted at the Periodontology Department of University Dental Clinic in Cracow, Poland. The study protocol has been published by Wozniak et al. [9]. The study was performed in accordance with the Helsinki Declaration. All the participants gave informed consent to participate in the study. Official approval from the Jagiellonian University Ethics Committee was obtained (No. KBET/106/B/2011). The participants were enrolled during the dental appointments. The trial design is presented in the diagram (Figure 1). After the trial, patients were referred for follow-up dental care or treatment as needed.

## 2. Materials and Methods

### 2.1. Participants

Sixty volunteers aged 18 or more were recruited into the study. A total of 98 subjects who complained of intra-oral halitosis were initially screened, which resulted in the inclusion of 60 participants (30 women, 30 men) aged 25–65, who met the inclusion criteria. None of the participants took any antibiotics for the past 6 months and no non-steroid anti-inflammatory drugs nor corticosteroids within the last 3 months. They had to be nonsmokers, free from caries, inflammatory lesions of the oral mucosa and gingivitis or periodontitis, with the presence of more than 19 teeth. Pregnancy, any chronic disease, pharmacologic or radiologic therapy in the 6 months prior to the study were also added to the exclusion criteria. The study population was divided into two groups: group A and group B. Each person who was included in the study was addressed to the groups according to the order: the first patient qualified for the study was assigned to group A and the next to group B, etc.

### 2.2. Data Collection

Data were collected during scheduled dental appointments in the morning (between 7:30 a.m. and 10:30 a.m.). Demographics and oral hygiene routines were recorded. The oral examination was performed, and intra-oral halitosis was investigated. Patients were asked not to eat, not to brush their teeth, nor to use scented products on the appointment day. Participants were also asked not to drink alcohol or eat any spicy food a day before. All measurements were performed by the same qualified examiner, who was blind to the intervention group. The tongue coating was assessed according to the Miyazaki coating index [10].

### 2.3. Intra-Oral Halitosis Assessment

A single trained and calibrated odor judge performed the organoleptic evaluation using the Rosenberg scale. The examiner (AD) has participated in “Organoleptic Judge Course” at the University of the West of England (UK) prior the study. Subjects were asked to close their mouths for 60 s, not to swallow during this period and open their mouth afterward. The judge immediately recorded the odor rating: 0 = no odor; 1 = doubtful slight malodor; 2 = slight malodor; 3 = moderate malodor, 4 = strong malodor, 5 = very strong malodor. After the organoleptic evaluation, samples of breath were assessed by a gas chromatography Oral Chroma device (Abimedical, Abilit Corporation, Osaka City, Japan). The concentrations of hydrogen sulfide (H_2_S), methylmercaptan (CH₃SH) and dimethyl sulfide ((CH₃)₂S) were measured in parts per billion (ppb). The total sum of all three Voltaire Sulfur Compounds concentrations (VSC) higher than 125 ppb was considered as intra-oral halitosis.

### 2.4. Intervention

Hygiene kits in paper bags were prepared and administrated to volunteers by another researcher on the same day the investigation proceeded. One kit contained a toothbrush (medium, Colgate-Palmolive Company, Grabetsmattweg, Switzerland), mouthwash (Meridol Halitosis, GABA, Therwil, Switzerland), Meridol Halitosis tooth and tongue gel (GABA, Therwil, Switzerland) and tongue scraper (Meridol Halitosis, GABA, Switzerland), and the other kit contained a toothbrush (medium, Colgate-Palmolive Company, Switzerland) and a toothpaste (Colgate Cavity Protection, Colgate-Palmolive Company, Switzerland). Instructions on the use of mouthwash, tongue scraper, tooth and tongue gel and toothbrushing were provided accordingly. In order to standardize baseline measurements and avoid interference from the presence of dental plaque, one day before each experimental phase, all volunteers had professional supragingival plaque removal performed. 

**Figure 2:**
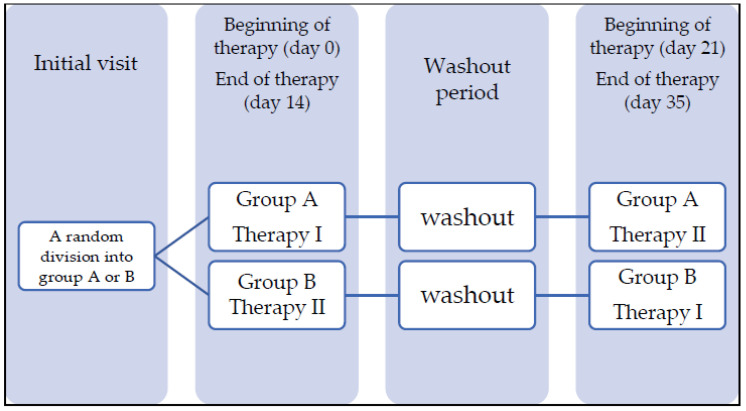
The study design.

A blind comparison of 60 volunteers divided into two cross-over groups was performed after each experimental period of 14 days. In each period, every volunteer performed one of the oral hygiene protocols: tooth brushing (Therapy I—TI) or tooth brushing, tongue scraping and gel placement and using a mouthwash (Therapy II—TII). Group A started with Therapy I, and group B started with Therapy II. Each 14-day experimental period was followed by a 7-day wash-out interval, and then Therapies I and II were switched within the groups for the next 14-day experimental period (Figure 2). The 7-days long wash-out was introduced as in other studies of this type [3,8]. Inbetween the experimental periods, the subjects maintained the standard oral hygiene they were performing during the pre-study period. They were using Colgate Cavity Protection toothpaste and were instructed that during the study, they should not use other oral hygiene products, including dental floss (this did not apply to the wash-out phase) or undergo any other dental visits. Compliance was assessed after each experimental period. Therapy I included tooth brushing twice a day for 2 min with toothpaste (Colgate Cavity Protection) for 14 days. Therapy II included brushing teeth for 2 min, cleaning the tongue for 10 s and rinsing the mouth with 10 ml of mouthwash twice a day for 60s. Ingredients of the products used in the study are as follows:

Meridol Halitosis mouthwash; active ingredient: zinc lactate (0.14%), other ingredients: aqua, xylitol, propylene glycol, PVP, PEG-40 hydrogenated castor oil, zinc lactate, olaflur; Aroma: stannous fluoride, sodium saccharin, CI 42051; Manufacturer: GABA, Switzerland; Packaging: 400 mL bottle.

Meridol Halitosis tooth and tongue gel; active ingredient: zinc lactate (0.14%), other ingredients: aqua, sorbitol, glycerin, hydrated silica, hydroxyethylocellulose, sodium gluconate; Aroma: Peg-3 tallow aminopropylamino, cocamidopropyl betaine, zinc lactate, stannous fluoride, potassium hydroxide, saccharine, hydrochloric acid, limonene, CI 74; Manufacturer: GABA, Switzerland; Packaging: 75 mL tube.

Colgate Cavity Protection toothpaste; active ingredient: sodium monofluorophosphate (0.76%), other ingredients: dicalcium phosphate dihydrate, aqua, glycerin, sodium lauryl sulfate, cellulose gum, tetrasodium pyrophosphate, sodium saccharin, sodium fluoride; Aroma (>100 ppm): anethole, carvone, mentha piperita (peppermint) oil, mentha viridis (spearmint) leaf oil, menthol; Manufacturer: Colgate-Palmolive Company, Switzerland; Packaging: 75 mL tube.

Patients were asked about any side effects or discomfort during each appointment and none were reported.

### 2.5. Statistical Methods

All the calculations were performed in Statistica 6.0 for Windows (Statistic for Windows, Statsoft, Tulsa, OK, USA, 2001). With the significance level set to 0.05 and the power set to 0.9, the calculated minimum required sample size was 28 subjects per group. The normality of the distribution of variables was tested using the Shapiro–Wilk test. The variables’ differences were analyzed by the Chi-square test. The Wilcoxon signed-rank test was used to compare the two paired samples. The effect size was assessed by the Mann–Whitney *U* test (0.139). The *t*-test was used to check whether any carry-over effect in the cross-over test model exists.

## 3. Results

### 3.1. Side Effects and Safety Monitoring

No side effects were tracked, and no rescue therapy was required in any of the patients throughout the study. All patients enrolled in the study were followed by adequate dental treatment and follow-up after data collection. All enrolled volunteers completed the study.

### 3.2. Study Population

The description of the study population is presented in Table 1. There were no statistically significant differences in terms of gender and age between groups A and B observed. 

### 3.3. Check for the Presence of a Carry-over Effect

As the primary goal of this cross-over study is to determine whether there are significant differences between Therapy I and Therapy II, the presence of carry-over effect was checked.

Based on the obtained results, the following hypotheses were subjected to the *t*-test:

**H0:**  *There is no carry-over effect between Therapy I and Therapy II.*

**H1:**  *There is a carry-over effect between Therapy I and Therapy II.*

No significant carry-over effect between therapies for VSC (*p*-value 0.26), CH3SH (*p*-value 0.62, H_2_S (*p*-value 0.08), (CH_3_)_2_SH (*p*-value 0.49), tongue coat (*p*-value 0.65) and organoleptic score (*p*-value 0.11) was observed.

### 3.4. Comparison of TI and TII Therapies

Table 2 presents halitosis results for TI and TII therapies. The last column contains data on the dynamics of changes between the beginning (TB) and the end (TE) of each therapy.

Therapy II ensured a decrease in CH_3_SH level at the end of therapy compared to its beginning by 52.65% (*p*-value 0.07), and in Therapy I, an increase of 16.03% (*p*-value 0.74) was observed. Comparing the effects of TI and TII therapies, in the case of H_2_S, Therapy II reduced the level of H_2_S by 50.69% (*p*-value 0.00), and Therapy I caused a decrease by 16.59% (*p*-value 0.42). TI achieved a lower level of (CH_3_)_2_S than the TII in all analyzed periods. The total VSC concentrations were lower for the TII therapy than for the TI therapy in all periods. TI therapy provided a decrease of 5.53% (*p*-value 0.76) in VSC concentration, while TII therapy achieved a decrease of 51.26% (*p*-value 0.00) . TI reduced the organoleptic assessment by 15.66% (*p*-value 0.01) and the TII by 21.69% (*p*-value 0.00).

The comparison of the tongue coating for the two Therapies (I and II) are presented in Table 3.

In the course of TI therapy, the amount of raid on the tongue decreased by 10.87% (*p*-value 0.09), and in TII, it decreased by 27.65% (*p*-value 0.00).

There were statistically significant differences for tongue coating between the begin-ning to end of the therapy TII (*p*-value 0.00) and organoleptic evaluation between the beginning to end of the TI therapy (*p*-value 0.00) . Also H_2_S between the beginning to end of the TII therapy and VSC between the beginning to the end of the TII therapy results were statistically significant (*p*-value 0.00). There were also statistically significant differences for organoleptic evaluation score between the beginning to the end of the TII therapy (*p*-value 0.00), H_2_S between the end of the first and second therapy (*p*-value 0.05) and VSC between the end of the TI and TII therapies (*p*-value 0.00). There was a significant positive correlation between organoleptic scores and Oral Chroma measure-ments (*p* < 0.05).

Summarizing the results:

- H_2_S: there was a decrease in the value at the end of the first therapy compared to its beginning by 36.10%, and by 38.39% in the case of the corresponding periods of the second therapy;
- Organoleptic evaluation: there was a decrease in the value at the end of the first therapy compared to its beginning by 18.55%, and by 19.38% in the case of the same periods of the second therapy;
- A coat on the tongue: a decrease in the value was noted at the end of the first therapy compared to its beginning by 20.33%, and by 17.83% in the case of the same periods of the second therapy.

## 4. Discussion

Many publications report that halitosis is an oral health problem in the general population that is underestimated [11,12,13]. The origin of halitosis is mainly associated with intra-oral factors due to humidity and higher temperature in the oral cavity, which creates a nature for the development of the disease [14,15,16]. This condition is found to be more observed in older people. Sometimes it is not recognized by the patients themselves [10]. Intra-oral halitosis prevalence in males and females and in relation to patients’ age was studied by Nadanovsky et al. [17]. Authors rated halitosis levels higher in men than women. It was the highest at the age of 20 in both sexes. In another study by Villa et al., the results suggested that individuals over the age of 13 and mostly females show common halitosis symptoms [18]. However, there is no consensus on whether the prevalence of halitosis is greater in males or females [2,17,18]. In our study, there were no statistically significant differences in terms of gender and age between groups A and B observed, which was important to preserve the unity of the groups for the cross-over study design. The relation of halitosis with periodontal diseases was also studied by many research groups. Deutscher et al. conducted a systematic review to analyze halitosis in patients with periodontal diseases [19]. The aim of their review was to explore whether professional cleaning or periodontal therapy would treat halitosis. They concluded that periodontal therapy reduces halitosis in patients who suffer from periodontal diseases. There are also other publications showing the correlation between periodontal diseases and halitosis [20,21,22,23]. Moreover, smoking is reported to be in association with halitosis [13,24]. It is suggested that smoking has a pathological effect on the periodontium and also alters the inter balance of oral microflora [25].

The primary goal of this cross-over study was to determine whether there are significant differences between Therapy I and Therapy II on halitosis. The effect of tongue cleaning was studied in many publications. They report that tongue coating is a very strong factor for halitosis [14,26,27,28,29]. Allaker et al. investigated bacteria around the tongue surface and suggested they are associated with halitosis [13]. Authors demonstrated that inaccessible tongue surfaces that could not be cleaned sufficiently could significantly contribute to halitosis. Morita et al. studied the correlation between the volume of tongue coating and reported a positive correlation associated with halitosis [26]. Monea et al. analyzed the bacteria samples from the dorsal side of the tongue surface and concluded that harvested microflora could have been the factor influencing the oral halitosis [28]. Yoneda et al. studied the relationship between the parameters associated with oral halitosis. Saliva samples were collected from halitosis patients, and the results of the analysis indicated that the β-galactosidase activity is a key factor for halitosis. The β-galactosidase activity was in positive correlation with malodor strength and with the degree of tongue coating observed [29]. Our study provided parallel results with the scientific evidence; in the course of Therapy I, the amount of raid on the tongue decreased by 10.87%, and in Therapy II, it decreased by 27.65%. There is a statistically significant decrease in tongue coating between the beginning and the end of Therapy II.

Winkel et al. investigated the effect of mouth rinse in halitosis treatment in a group of periodontally healthy patients [30]. A mouth rinse, including chlorhexidine, cetylpyridinium chloride and zinc lactate, was compared with a placebo solution without any active substance. The study resulted in a clear decrease in organoleptic scores of halitosis in the active mouth-rinsing group compared to the placebo group [30]. The authors concluded that the mouth rinse with active substance was effective in halitosis treatment. 

A randomized clinical trial to evaluate the long-term effects of zinc acetate and chlorhexidine diacetate mouth rinse (Zn/CHX) on intra-oral halitosis was carried by Ademovski et al. [31]. They found Zn/CHX mouth-rinsing to be effective in the treatment of halitosis. Aung et al. in 2015 conducted a clinical study comparing tooth brushing and mouth washing to tooth brushing and tongue cleaning for a period of one month. They concluded that tooth brushing could not decrease the halitosis, but when combined with mouth washing and tongue cleaning, it is effective in halitosis treatment [32]. Quirynen et al. published opposite conclusions [33]. They assessed the efficacy of several antiseptics in the prevention of bad morning breath and demonstrated that the sole mouth-rinsing two times a day without toothbrushing could be effective. Mouth-rinsing significantly reduced the bacterial load in the saliva and retarded the de novo plaque formation, but due to the limited oral hygiene during the experimental periods, bad breath parameters systematically improved [33]. In the study conducted by Wigger-Alberti et al., 250 ppm F(−) from amine fluoride/stannous fluoride (ASF) + 0.2% zinc lactate mouth rinse was compared to 0.12% chlorhexidine. They reported no statistically significant differences but mentioned the fewer side effects caused by the ASF product compared to the products containing CHX [34]. In 2019, Srisilapanan et al. published their results of a study testing the combination of toothpaste and mouth rinse containing 0.14% zinc lactate, and they reported that this combination reduced the three volatile gases and total VSC at all assessment times, which was similar to our results [8]. Our study revealed statistically significant differences between the beginning and end of the TII therapy for tongue coating, organoleptic evaluation, H_2_S levels and VSC ranges. The findings of our study indicate that complex approaches in the hygienic protocol, including tooth brushing, tongue scraping and zinc lactate-containing mouthwash, can be effective in halitosis treatment.

## Figures and Tables

**Table 1:** The description of the study population.

Quantity			Group		Total
			A	B	
Sex	Women	*N*	17	21	38
	Men	*N*	13	9	22
Total		*N*	30	30	60
Age		Mean	46.46	40.60	
		Minimum	25.00	18.00	
		Maximum	65.00	65.00	

**Table 2:** Comparison of intra-oral halitosis parameters between Therapies I and II.

Mean/Day	TB	TB + 30 min	TB + 1 day	TE	TB vs. TE	*p* value
CH_3_SH (ppb) (TI)	115.53	99.75	97.35	134.05 ppb	16.03%	0.74
CH_3_SH (ppb) (TII)	160.52	38.00	149.12	76.00 ppb	−52.65%	0.07
H_2_S (ppb) (TI)	585.17	457.38	433.23	488.07 ppb	−16.59%	0.42
H_2_S (ppb) (TII)	609.73	196.62	450.88	300.68 ppb	−50.69%	0.00
(CH_3_)_2_S (ppb) (TI)	32.92	51.63	35.33	67.44 ppb	104.88%	0.21
(CH_3_)_2_S (ppb) (TII)	67.44	87.72	51.03	92.22 ppb	36.74%	0.39
VSC_total (ppb)_ (TI)	914.14	804.30	683.75	863.59 ppb	−5.53%	0.76
VSC_total (ppb)_ (TII)	863.37	285.65	673.05	420.78 ppb	−51.26%	0.00
Organoleptic score (TI)	2.20	1.74	1.94	1.86	−15.66%	0.01
Organoleptic score (TII)	2.31	1.65	1.96	1.81	−21.69%	0.00

TB—Therapy Beginning, TE—Therapy End, TI—Therapy I, TII—Therapy II.

**Table 3:** The comparison of the tongue coating for the two Therapies I and II.

Mean/Day	TB	TE	TB vs. TE	*p* value
Tongue coating (TI)	6.90	6.15	−10.87%	0.09
Tongue coating (TII)	7.53	5.45	−27.65%	0.00

TB—Therapy Beginning, TE—Therapy End.

## Data Availability

The data presented in this study are available on request from the corresponding author. The data are not publicly available due to ethical restrictions.
